# Suppression of dynamin GTPase decreases α-synuclein uptake by neuronal and oligodendroglial cells: a potent therapeutic target for synucleinopathy

**DOI:** 10.1186/1750-1326-7-38

**Published:** 2012-08-14

**Authors:** Masatoshi Konno, Takafumi Hasegawa, Toru Baba, Emiko Miura, Naoto Sugeno, Akio Kikuchi, Fabienne C Fiesel, Tsutomu Sasaki, Masashi Aoki, Yasuto Itoyama, Atsushi Takeda

**Affiliations:** 1 Division of Neurology, Department of Neuroscience and Sensory Organs, Tohoku University Graduate School of Medicine, Sendai, Miyagi, 980-8574, Japan; 2 Department of Neuroscience, Mayo Clinic, 4500 San Pablo Road, Jacksonville, FL, 32224, USA; 3 Department of Neurology, Osaka University Graduate School of Medicine, Suita, Osaka, 565-0871, Japan; 4 National Center Hospital for Mental, Nervous, and Muscular Disorders, National Center of Neurology and Psychiatry (NCNP), Kodaira, Tokyo, 187-8502, Japan

**Keywords:** α-synuclein, Neuron, Oligodendroglia, Transmission, Inclusions, Endocytosis, Dynamin, Sertraline, Parkinson’s disease, Multiple system atrophy

## Abstract

**Background:**

The intracellular deposition of misfolded proteins is a common neuropathological hallmark of most neurodegenerative disorders. Increasing evidence suggests that these pathogenic proteins may spread to neighboring cells and induce the propagation of neurodegeneration.

**Results:**

In this study, we have demonstrated that α-synuclein (αSYN), a major constituent of intracellular inclusions in synucleinopathies, was taken up by neuronal and oligodendroglial cells in both a time- and concentration-dependent manner. Once incorporated, the extracellular αSYN was immediately assembled into high-molecular-weight oligomers and subsequently formed cytoplasmic inclusion bodies. Furthermore, αSYN uptake by neurons and cells of the oligodendroglial lineage was markedly decreased by the genetic suppression and pharmacological inhibition of the dynamin GTPases, suggesting the involvement of the endocytic pathway in this process.

**Conclusions:**

Our findings shed light on the mode of αSYN uptake by neuronal and oligodendroglial cells and identify therapeutic strategies aimed at reducing the propagation of protein misfolding.

## Background

Lewy bodies (LBs), which are the cardinal histological hallmark of Parkinson’s disease (PD), contain abnormal filamentous α-synuclein (αSYN) aggregates. In addition, a variety of other neurodegenerative diseases are associated with αSYN-positive lesions [1,2]. The presence of αSYN in LBs, Lewy neurites or glial cytoplasmic inclusions (GCIs) in PD and related disorders provides a conceptual link that has led to the use of the term ‘synucleinopathy’ to encompass these diseases [3-5]. Emerging evidence from genetic and biochemical studies demonstrates that abnormal αSYN aggregates directly contribute to neurodegeneration in PD and other synucleinopathies [6-10]. Because αSYN is enriched in the presynaptic nerve terminals and is mainly detected in the cytosolic and synaptosomal fractions, it has long been believed that αSYN exerts its physiological as well as pathogenic effects intracellularly [11,12]. However, accumulating evidence suggests that both monomeric and oligomeric αSYN can be secreted into the extracellular milieu, thereby affecting the normal physiological state of neighboring cells [13-17]. For example, *in vitro*-generated soluble αSYN oligomers can induce the transmembrane seeding of αSYN aggregation and can eventually lead to cell death [18,19]. Moreover, exogenous αSYN fibrils have been shown to induce perikaryal LB pathology, which can lead to synaptic dysfunction and neuronal cell death [20]. The existence of the transcellular spread of αSYN has also been verified by co-culture experiments and *in vivo* animal models, which showed that αSYN aggregates released from neuronal cells can be transferred to neighboring cells and form inclusion bodies [21-23]. Finally, the existence of an *in vivo* intercellular propagation of αSYN aggregates was supported by recent observations of LB-like inclusions in the grafted neurons of PD patients who had received transplants of fetal mesencephalic neurons more than a decade previously [24-26]. In addition to PD, the intercellular transmission of αSYN pathology can be assumed to be present in multiple system atrophy (MSA), in which widespread αSYN-positive GCIs are found in oligodendroglia, a type of brain cell that does not normally express αSYN [27-29]. Phenomenologically, the propagation theory is also attractive as an explanation for the hierarchical distribution of Lewy pathology in PD, a theory proposed by Braak and colleagues [30]. To understand how αSYN travels from cell to cell, the underlying mechanisms responsible for αSYN uptake and secretion must be elucidated. In this study, we provide evidence to support the functional role of dynamin-mediated endocytosis in the process of αSYN uptake by neurons and oligodendroglial cells. Furthermore, we propose therapeutic strategies aimed at reducing the propagation of protein misfolding in synucleinopathies.

## Results

### The characterization of recombinant α-synuclein

**Figure 1:**
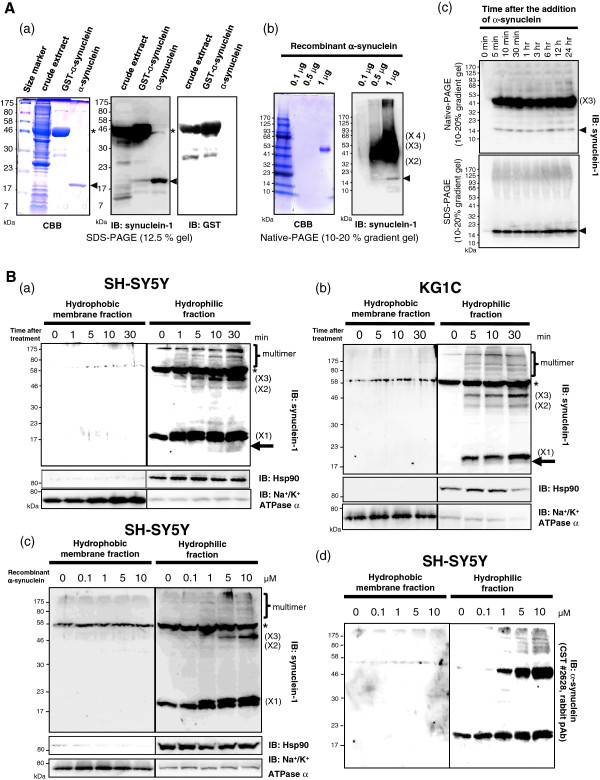
**Extracellularα-synuclein is internalized and assembled into SDS-stable oligomers in neuronal and oligodendroglial cells. A**, The characterization of recombinant αSYN. (**a**) The expressed GST-αSYN and tag-free αSYN were analyzed by CBB staining and western blotting with an anti-αSYN Ab (1 μg per lane). Upon IPTG induction, the transformed *E. coli* produced a GST-αSYN fusion protein that migrated at 44 kDa under denaturing conditions (*asterisk*). Following the removal of the GST-tag, monomeric αSYN was detected, which corresponded to a molecular mass of 18 kDa (*arrowhead*). An immunoblot using an anti-GST antibody did not detect any GST-αSYN after the removal of the GST moiety. (**b**) In the native condition, the absolute majority of recombinant αSYN migrated to approximately 54 kDa, corresponding to trimeric (X3) αSYN as well as some monomers (*arrowhead*) and oligomers (X2 and X4)/multimers. (**c**) Recombinant αSYN in cell-free culture medium did not self-assemble into SDS-stable oligomers after 24 hours at 37°C. **B**, Extracellular αSYN was incorporated and assembled into oligomers in neuronal and oligodendroglial cells. SH-SY5Y (**a**) and KG1C (**b**) cells were exposed to 5 μM αSYN for the indicated amount of time, and the cells were then subjected to fractionation and αSYN immunoblot analysis (50 μg lysate per lane). One minute after the addition of αSYN, monomeric αSYN (X1) was incorporated, and the amount of αSYN increased thereafter mainly in the hydrophilic fraction. In parallel, the SDS-resistant dimeric/trimeric αSYN (X2-X3), as well as the multimers and truncated fragments (*arrow*), gradually appeared in the hydrophilic fractions. Hsp90 and Na^+^/K^+^ ATPase α were used as markers for the cytosol and the plasma membrane, respectively. (**c**) A dose-dependent increase in intracellular monomeric (X1) and oligomeric (X2-X3) αSYN was observed mainly in the hydrophilic fractions prepared from the cells exposed to varying concentrations of recombinant αSYN (0–10 μM) for 24 hours. An *asterisk* indicates the non-specific band. Representative blots from three independent experiments are presented.

Ectopically expressed proteins were collected from crude bacterial lysates and analyzed by sodium dodecyl sulfate-polyacrylamide gel electrophoresis (SDS-PAGE) followed by Coomassie brilliant blue (CBB) staining and immunoblotting with an anti-αSYN antibody (Ab). Upon isopropyl β-D-1-thiogalactopyranoside (IPTG) induction, the BL21(DE3)pLysS *E. coli* was transformed with pGEX6P-1/αSYN, which produced a GST-αSYN fusion protein that migrated at 44 kDa under denaturing conditions (Figure 1A, *asterisk*). After enzymatic removal of the GST-tag, monomeric αSYN was detected at a molecular mass of approximately 18 kDa, which was larger than the predicted value of 14 kDa (Figure 1A (a), *arrowhead*). Immunoblot analysis, using the anti-GST antibody, did not detect any remaining GST-αSYN after the removal of the GST moiety. The slow gel mobility may be attributed to the weak binding of SDS due to the highly acidic C-terminal sequence of αSYN [31]. However, under native conditions, the majority of the recombinant αSYN migrated to a position corresponding to approximately 54 kDa, which was assumed to be composed mainly of trimers as well as a few monomers and oligomers/multimers (Figure 1A (b)). It is important to note that the recombinant αSYN did not self-assemble into SDS-resistant, soluble oligomers after 24 hours at 37°C in the cell-free culture medium (Figure 1A (c)). There was no visible band in the PBS washing buffer that flowed through the column, as revealed by CBB staining and αSYN immunoblotting (data not shown). 

### **Extracellular** α**-synuclein was incorporated and assembled into oligomers in neuronal and oligodendroglial cells**

To elucidate how αSYN is internalized into cells, human SH-SY5Y neuronal and KG1C oligodendroglial cells [32,33] were exposed to 5 μM αSYN for the indicated amount of time, and then subjected to fractionation and immunoblot analysis (Figure 1B (a) and (b), respectively). The KG1C cells used in the present study were confirmed to express oligodendroglial markers, including CNPase and myelin basic protein [34]. Interestingly, only one minute after the addition of αSYN, monomeric αSYN (X1) had been incorporated and then continued to increase mainly in hydrophilic fraction. In parallel, the SDS-stable dimeric/trimeric αSYN (X2-X3), as well as the multimers and truncated fragments (*arrow*), also gradually appeared in hydrophilic fraction. Similarly, a dose-dependent increase in the intracellular monomeric and oligomeric forms of αSYN was observed in the SH-SY5Y cells that were exposed to different concentrations of recombinant αSYN (0–10 μM) for 24 hours (Figure 1B (c)). To clearly identify the high-molecular-weight (HMW) αSYN species, the same blot was stripped and reprobed with the rabbit polyclonal αSYN Ab (#2628, Cell Signaling Technology) to eliminate the non-specific band detected by the anti-synuclein-1 Ab (Figure 1B (d)). In addition, the amount of monomeric αSYN in the hydrophilic fractions of exposed cells was quantitatively measured by densitometric analysis (Additional file 1: Figure S1A (a)-(c). Similarly to the untagged αSYN, the GST-αSYN added to the culture medium was time-dependently incorporated into SH-SY5Y cells and formed GST-immunopositive oligomers and an HMW smear mainly in the hydrophilic fraction, demonstrating that extracellular αSYN was internalized and oligomerized in the exposed cells (Additional file 1: Figure S1B). In mammals, the SYN family consists of three members (α-,β-,γ-), and all SYN genes are well conserved across species [35]. Importantly, we found that αSYN was exclusively internalized into cells compared to β-synuclein (βSYN) and γ-synuclein (γSYN). In addition, the familial PD-linked A30P and A53T mutations of αSYN enhanced the buildup of the SDS-stable HMW species when compared to the wt-αSYN (Additional file 2: Figure S2). It is important to note that αSYN (10 μM) exposure for up to 10 days did not result in any changes in the cellular morphology or growth retardation (data not shown).

### The formation of cytoplasmic inclusions in neuronal and oligodendroglial cells exposed to α-synuclein

**Figure 2:**
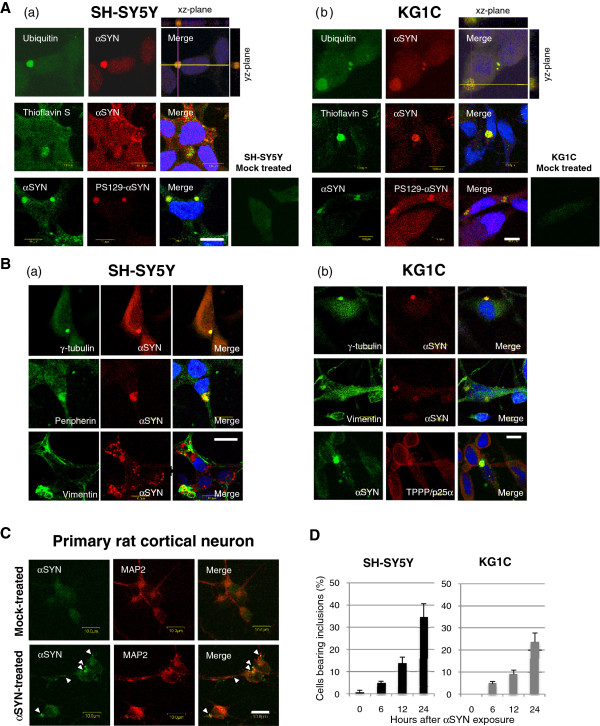
**The formation of cytoplasmic inclusions in neuronal and oligodendroglial cells exposed toα-synuclein. A**, Large perikaryal inclusions as well as small aggregates were observed in the SH-SY5Y (**a**) and KG1C (**b**) cells exposed to 5 μM recombinant αSYN for 24 hours. The αSYN-positive inclusions and small aggregates were positive for ubiquitin and thioflavin S staining, whereas only the large inclusions co-immunostained positively for phosphorylated serine 129-αSYN. Neither αSYN-positive inclusions nor small aggregates were detected in cells treated with the column flow-through. The crossed lines indicate the positions of the xz- and yz-planes. **B**, Double-immunolabeling studies demonstrated that the juxtanuclear large inclusions co-localized with γ-tubulin, peripherin, and/or vimentin, which are known markers of the aggresome (**a** and **b**). The αSYN-positive inclusion bodies in the KG1C cells **(b)** were co-localized with TPPP/p25α, a known marker for GCI in MSA. **C**, The formation of αSYN-positive cytoplasmic inclusions (*arrowhead*) was also confirmed in primary rat cortical neurons treated under the same condition. No visible αSYN-positive inclusions were detected in the mock-treated cells. **D**, After αSYN exposure, the incidence of perinuclear inclusions in both the SH-SY5Y and KG1C cells increased in a time-dependent manner, reaching 35% and 24%, respectively, at 24 hours. Data are expressed as the mean ± standard errors. The immunostaining was performed three times and exhibited consistent results. Scale bar: 10.0 μm.

Immunocytochemistry showed that large perinuclear inclusions, as well as small aggregates, were observed in the SH-SY5Y and KG1C cells exposed to 5 μM recombinant αSYN for up to 24 hours. In both cell lines after treatment, the αSYN-positive large inclusions and small aggregates were positive for ubiquitin and thioflavin S staining, but only the large inclusions were co-immunostained for serine 129-phosphorylated αSYN in both cells. Neither αSYN-positive inclusions nor small aggregates were detected in control cells treated with the column flow-through (Figure 2A (a) and (b)). The spatial co-localization of ubiquitin and the αSYN-positive inclusions was confirmed by orthogonal reconstructions from confocal z-stacks in the xz- and yz-planes. Furthermore, the double-immunolabeling studies, depicted in Figure 2B (a) and (b), demonstrated that the large juxtanuclear large inclusions were not only co-localized with γ-tubulin and peripherin but were also enveloped within a vimentin cage, suggesting that the molecular machinery controlling aggresomal formation may contribute to their biogenesis [2]. Of note, the αSYN-positive inclusion bodies in the KG1C cells were co-localized with tubulin-polymerization-promoting protein/p25α (TPPP/p25α), a known marker of GCI in the affected brain lesions of MSA patients [34,36,37]. The formation of αSYN-positive cytoplasmic inclusions was also confirmed in primary rat cortical neurons treated under the same condition, suggesting that these results were not a cell line-specific phenomenon (Figure 2C). The percentage of perinuclear inclusion-bearing SH-SY5Y and KG1C cells increased in a time-dependent manner, reaching 35% and 24%, respectively, at 24 hours (Figure 2D). 

### Lysosomal inhibition enhanced α-synuclein oligomer formation and impaired autophagic flux

**Figure 3:**
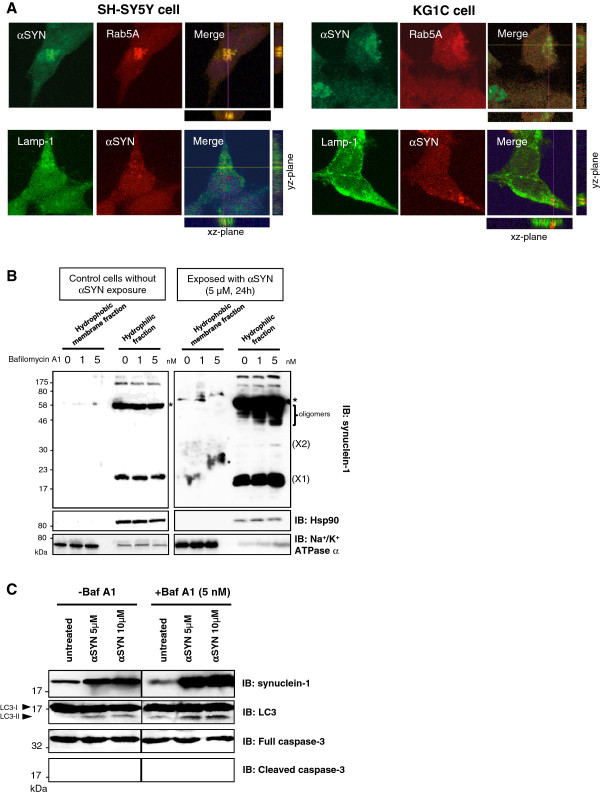
**Lysosomal inhibition facilitates α-synuclein oligomer formation and impairs autophagic flux. A**, A portion of the αSYN-positive aggregates found in the αSYN-treated SH-SY5Y cells (5 μM recombinant αSYN for 24 hours) showed partial co-localization with markers of early endosomes (Rab5A) and lysosomes (Lamp-1). The crossed lines indicate the positions of the xz- and yz-planes. Immunostaining was performed three times and exhibited consistent results. Size bar: 10.0 μm. **B**, Pretreatment of the cells with bafilomycin A1 (0–5 nM) increased the intracellular accumulation of the αSYN oligomers (X2 and higher) in a dose-dependent manner. Furthermore, this increase was mainly observed in the hydrophilic fractions of the αSYN-exposed cells, whereas the level of the αSYN monomer (X1) was unchanged. Fifty micrograms of lysates were analyzed by immunoblotting and the blot was probed with an anti-αSYN Ab. Hsp90 and Na^+^/K^+^ ATPase α were used as markers for the cytosol and the plasma membrane, respectively. The *asterisk* indicates the non-specific band. **C**, In the αSYN-exposed SH-SY5Y cells (5 μM recombinant αSYN for 24 hours), bafilomycin (5 nM) treatment augmented the induction of the LC3-II protein, whereas no cleaved fragments of caspase-3 were detected. Representative western blots from three independent experiments are presented.

The αSYN-positive aggregates in the cells exposed to recombinant αSYN were partially co-localized with the markers for early endosomes (Rab5A) and lysosomes (Lamp-1) (Figure 3A). To confirm the role of the intact lysosomal system in the degradation of incorporated αSYN, SH-SY5Y cells were treated with the vacuolar H^+^ ATPase inhibitor bafilomycin A1 (0–5 nM) together with 5 μM αSYN for 24 hours. Following the fractionation process, all samples were analyzed by western blot analysis (Figure 3B). As shown by the immunoblotting, bafilomycin treatment increased the intracellular accumulation of HMW αSYN oligomers in the hydrophilic fraction of αSYN-exposed cells in a dose-dependent manner. In contrast, bafilomycin treatment did not affect the level of endogenous αSYN in the control cells lacking αSYN exposure. In the αSYN-treated SH-SY5Y cells, co-treatment with bafilomycin augmented the induction of LC3-II protein, which is indicative of an increase in macroautophagy and autophagosome formation. Under this condition, we could not detect caspase-3 cleavage, a hallmark of apoptosis (Figure 3C). Hence, lysosomal inhibition impairs the autophagic flux in αSYN-treated neuronal and oligodendroglial cells.

### The inhibition of dynamin GTPases decreased α-synuclein internalization by neuronal and oligodendroglial cells

**Figure 4:**
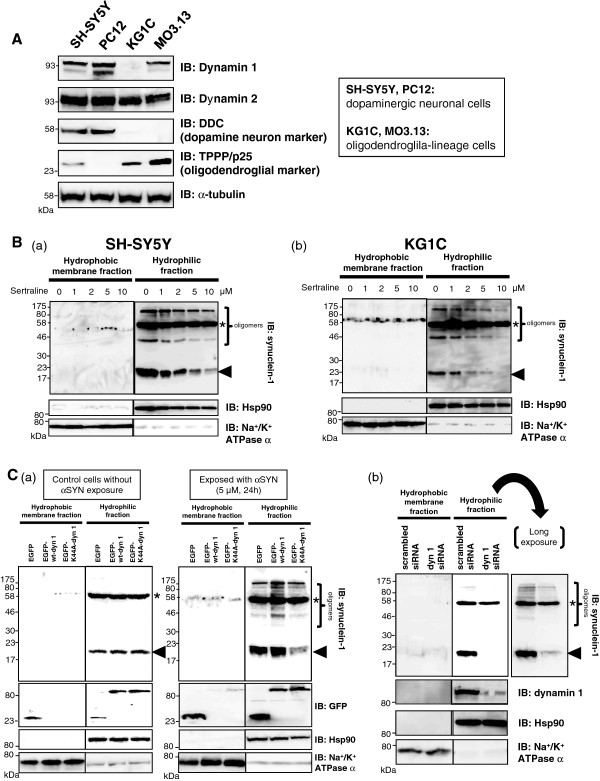
**The inhibition of dynamin GTPases decreases the internalization ofα-synuclein by neuronal and oligodendroglial cells. A**, The expression profile of various dynamin isoforms across neuronal and oligodendroglial cells. Dynamin 2 was widely expressed in both types of cells, whereas dynamin 1 was strongly expressed in dopaminergic neuronal cells. However, the expression of dynamin 1 was weak (MO3.13) or absent (KG1C) in cells of the oligodendroglial lineage. Equal loading was confirmed using α-tubulin as control. **B**, The pharmacological inhibition of dynamin GTPase activity via sertraline treatment (0–10 μM for 30 min) dose-dependently prevented both monomeric (*arrowhead*) and oligomeric αSYN accumulation in SH-SY5Y (**a**) and KG1C (**b**) cells. Both cells were treated with 5 μM αSYN for 30 min with different concentrations of sertraline, as indicated. The control cells were treated with solvent (0.1% DMSO) alone. Fifty micrograms of each lysate was analyzed by immunoblotting, and the blot was probed with an anti-αSYN Ab. Hsp90 and Na^+^/K^+^ ATPase α were used as markers for the cytosol and the plasma membrane, respectively. The *asterisk* indicates the non-specific band. **C**, Consistent with the results shown in Figure 4B, the inhibition of dynamin 1 through the use of either the K44A DN mutant (**a**) or siRNA (**b**) resulted in a marked reduction of internalized αSYN in SH-SY5Y cells. Cells were treated with 5 μM αSYN (for 30 min) 48 hours after either the DN-dynamin1 transfection or the siRNA silencing of dynamin 1. Note that the αSYN monomer and the SDS-stable HMW oligomers were increased in cells overexpressing wild-type dynamin 1. Representative immunoblots from three independent experiments are shown.

The internalization of αSYN oligomers is thought to be mediated by endocytosis because K44A dynamin 1, a dominant-negative mutant that blocks endocytic vesicle formation, was previously shown to reduce the number of incorporated αSYN aggregates in COS-7 cells and primary astrocytes [21,38,39]. The enhanced endocytosis of preformed fibrils of αSYN in response to the addition of wheat germ agglutinin (WGA) was shown to increase the extent of pathology in cultured neuronal cells [20]. Lee and his colleagues have shown that monomeric αSYN can be internalized into cells without endocytosis, whereas aggregated forms enter cells via endocytosis [19,39]. Complementarily, the inhibition of endocytosis decreased the cellular uptake of αSYN *in vitro* and *in vivo*[22]. The involvement of the endocytic process during αSYN internalization is further supported by a previous proteomic analysis showing that microglial activation, secondary to the internalization of aggregated αSYN, requires clathrin, which plays major roles in endocytosis and vesicular trafficking [40]. Of the three dynamin isoforms identified thus far, dynamin 1 and dynamin 2 are mainly involved in clathrin-mediated endocytosis [41]. Dynamin 1 is highly expressed in the nervous system, whereas dynamin 2 is ubiquitously expressed in all tissues [42,43]. To investigate the functional roles of dynamin GTPases in the incorporation of αSYN, we examined the expression profile of dynamin isoforms in neuronal and oligodendroglial cells (Figure 4A). As shown by immunoblotting, dynamin 2 was widely expressed in both types of cells, whereas dynamin 1 was strongly expressed only in the dopaminergic neuronal cells. However, the expression of dynamin 1 was weak (MO3.13) or absent (KG1C) in cells of the oligodendroglial lineage. It is known that several selective serotonin reuptake inhibitors (SSRIs) also inhibit dynamin GTPases, which suggests the influence of a class effect. Among the dynamin inhibitors thus far examined, sertraline (Zoloft®), a second-generation SSRI, exhibited the strongest effect against both dynamin 1 and 2 [41,43]. We found that the pharmacological inhibition of dynamin GTPase activity by sertraline treatment (50 mM stock in DMSO with a working concentration of 0–10 μM, Sigma) prevented αSYN uptake by SH-SY5Y and KG1C cells in a dose-dependent manner (Figure 4B (a) and (b), respectively). Both cells types were treated with 5 μM αSYN for 30 min with varying concentrations of sertraline as indicated. Consistent with this result, the inhibition of dynamin 1 through the use of a K44A dominant-negative (DN) mutant or small interference RNA (siRNA) resulted in a considerable decrease in αSYN incorporation into SH-SY5Y cells (Figure 4C (a) and (b), respectively). In addition, both αSYN monomers and SDS-stable HMW oligomers were increased in the cells overexpressing wild-type (wt) dynamin 1. The control experiment, which was not exposed to recombinant αSYN, demonstrated that dynamin 1 manipulation did not affect the expression level of endogenous αSYN in the SH-SY5Y cells. 

### Sertraline treatment inhibited the neuron-to-neuron and neuron-to-oligodendroglia transmission of α-synuclein in co-culture models

**Figure 5:**
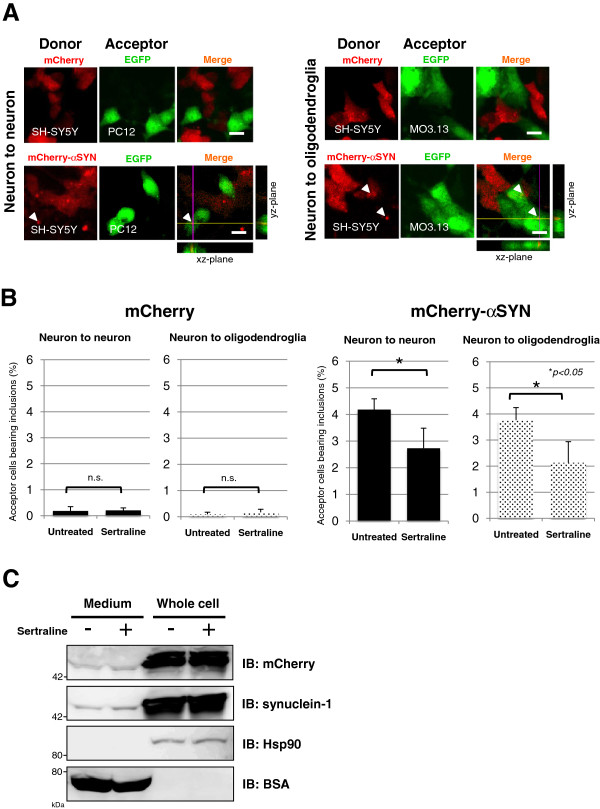
**Sertraline inhibits the neuron-to-neuron and neuron-to-oligodendroglia transmission ofα-synuclein in co-culture models. A**, SH-SY5Y cells overexpressing mCherry or N-terminally mCherry-tagged αSYN (donor cells) were co-cultured with PC12 neuronal or MO3.13 oligodendroglial cells stably expressing EGFP (acceptor cells) in the presence or absence of 10 μM sertraline. Vehicle-only-transfected cells were treated with solvent (0.1% DMSO) only. The mCherry fluorophore was used to trace the donor-derived αSYN protein. After 72 hours of co-culture, the transmission of mCherry-αSYN from the donor cells to the acceptor PC12 as well as MO3.13 cells was detected, confirming the uptake of mCherry-αSYN secreted from the donor cells. The arrowhead indicates the transferred mCherry-αSYN-positive aggregates in the acceptor cells. The crossed lines indicate the positions of the xz- and yz-planes. **B**, A co-culture experiment using donor cells expressing mCherry-αSYN showed that the percentage of acceptor cells with inclusions were 4.2% (PC12) and 3.8% (MO3.13), which is much higher than the percentage of inclusion-positive cells (under 0.2% in both cell lines) co-cultured with mCherry-expressing donor cells. The addition of sertraline to the culture medium reduced the incorporation of mCherry-αSYN into the acceptor cells (*p < 0.05), whereas this effect was not observed in the acceptor cells that were co-cultured with the mCherry-expressing donor cells. To quantify the mCherry-positive inclusions in the acceptor cells, the total number of cells containing aggregates was counted per 250–300 cells within five randomly selected fields. From this value, the percentages of cells with red-fluorescent inclusions were calculated. Pooled data from four independent experiments were statistically analyzed. Data are presented as the mean ± standard errors. **C**, Western blot analysis demonstrated that sertraline treatment did not affect the secretion of the mCherry-αSYN from the donor SH-SY5Y cells. BSA and Hsp90 were used as markers of the culture medium and the cytosolic proteins, respectively. Each immunoblot was performed at least three times and the replicates yielded similar results.

To test whether sertraline could prevent the cell-to-cell spread of αSYN, we performed co-culture experiments. For this purpose, SH-SY5Y cells overexpressing N-terminal mCherry-tagged αSYN (donor cells) were co-cultured with PC12 neuronal or MO3.13 oligodendroglial cells stably expressing Enhanced Green Fluorescence Protein (EGFP, acceptor cells), in the presence or absence of 10 μM sertraline. The mCherry fluorophore was used to trace the donor-derived αSYN protein. After 72 hours of co-culture, the transmission of mCherry-αSYN from the donor cells to the acceptor PC12 and MO3.13 cells was detected, confirming the uptake of mCherry-αSYN secreted from donor cells (Figure 5A). The spatial distribution detail of the mCherry fluorescence in donor cells was confirmed by orthogonal reconstructions from confocal z-stacks in the xz- and yz-planes. Co-culture experiments using donor cells expressing mCherry-αSYN showed that the percentage of acceptor cells with inclusions were 4.2% (PC12) and 3.8% (MO3.13), which was much higher than the percentage of the inclusion-positive cells (under 0.2% in both cell lines) from the co-culture with mCherry-expressing donor cells (Figure 5A and B). The addition of sertraline to the culture medium reduced the amount of incorporated mCherry-αSYN in the acceptor cells, whereas this effect was not observed in the acceptor cells co-cultured with mCherry-expressing donor cells (Figure 5B). We confirmed that the sertraline treatment did not affect the secretion of mCherry-αSYN from the donor SH-SY5Y cells (Figure 5C). It should be noted that the extracellular mCherry-αSYN released into culture media seems to be significantly lower than the recombinant αSYN used in the previous experiments. We hypothesize that this is the reason for the relatively low internalization of mCherry-αSYN in the acceptor cells of co-cultures compared to the level of inclusion formation in the cells exposed to recombinant αSYN. Together, these results suggest that the endocytic process contributes significantly to the neuronal as well as oligodendroglial uptake of αSYN.

## Discussion

Although αSYN is generally located in the cytoplasm, several studies have demonstrated that αSYN has an affinity for phospholipids and vesicles [44,45]. In addition, αSYN is known to be delivered to the plasma membrane through its interactions with the endoplasmic reticulum (ER)-Golgi secretory pathway [46]. Furthermore, increasing evidence has suggested that nanomolar concentrations of αSYN can be found in neuronal culture medium, as well as in body fluids such as plasma and cerebrospinal fluid [47,48]. The secreted monomeric and oligomeric forms of αSYN were shown to re-enter neighboring cells, resulting in various cytotoxic effects, such as the production of reactive oxygen species, the generation of a glial cell inflammatory response [18,21], and synaptic malfunction [20]. In the present study, we showed that, in cultured neuronal and oligodendroglial cells, the incorporated αSYN oligomers were assembled into SDS-stable HMW oligomers and could form LB- and GCI-like cytoplasmic inclusions. Except for KG1C cells, which do not express endogenous αSYN, it is possible that the internalized recombinant αSYN may be entangled with endogenous αSYN, and the assembled HMW oligomers in the SH-SY5Y neuronal cells may consist of both endogenous and recombinant αSYN. Consistent with a previous study, we confirmed that most of the internalized αSYN was located within the intracellular soluble fraction rather than the membrane fraction, indicating that extracellular αSYN rapidly crosses the cellular membrane [18]. Furthermore, wild-type αSYN has been shown to be degraded by lysosomes through chaperone-mediated autophagy, and decreased lysosomal function has also been observed in PD patients [49-52]. In agreement with this result, we detected internalized αSYN in the endosomal compartment, where it was eventually degraded by the lysosomes. Our data showed that lysosomal inhibition impairs the autophagic flux in αSYN-treated neuronal and oligodendroglial cells. This observation strongly suggests that proper lysosomal machinery is required for the clearance of the incorporated αSYN oligomers and is therefore indispensable for the maintenance of cellular homeostasis.

In addition to the Lewy pathology in PD, the presence of oligodendrocytic αSYN deposition, i.e., GCI, in MSA has attracted a significant amount of attention [29,53-55]. However, regarding the pathogenesis of MSA, it remains unclear why αSYN accumulates in an oligodendrocytes, which do not normally express endogenous αSYN [28]. It is possible that αSYN expression may be upregulated and/or inefficiently degraded in affected oligodendrocytes. Alternatively, it is plausible that oligodendrocytes may actively take up αSYN molecules derived from neurons. Indeed, endocytosis regulatory proteins such as Rab5 and Rabaptin-5 are known constituents of GCIs [56]. It is also possible that an intrinsic protein, such as TPPP/p25α, may promote the aggregation of internalized αSYN within oligodendroglia [34,57,58]. Interestingly, the prion-like hypothesis supports the possibility that aberrant oligodendroglial expression of αSYN may have an ectopic origin. Our study provides the first concrete evidence that extracellular αSYN can be incorporated and assembled into HMW oligomers and inclusions that mimic GCIs in cultured oligodendrocytes. In protein-folding diseases, protein misfolding and aggregation are thought to follow a ‘seeding-nucleation’ mechanism [59-61], whereby misfolded αSYN is transmitted from a LB-bearing donor cell to a neighboring recipient cell and can act as a template for the conversion of native, unfolded αSYN into a β-sheet-rich conformation within the recipient cell. However, given that cultured oligodendroglial cells without inherent αSYN expression can import extracellular αSYN and form HMW oligomers, the existence of endogenous αSYN may not be a prerequisite for the buildup of αSYN aggregates in the recipient cells.

The precise mechanisms by which the intercellular transmission of αSYN occurs remain controversial. Lee and colleagues implicated exocytosis as a plausible mechanism for αSYN release from cultured neuronal cells because its release was effectively blocked under low-temperature conditions [39]. As brefeldin A, an inhibitor of ER-Golgi-dependent exocytosis, is ineffective at preventing the secretion of nascent αSYN secretion by MES cells, αSYN exocytosis is thought to rely on an unconventional exocytic pathway [62]. Intriguingly, the small GTPase Rab5, which is a known marker of early endosomes, is critical for the endocytosis of exogenous αSYN into neuronal cells [18]. In a yeast model, the A30P mutant αSYN was shown to bind the endocytic cargo-transport protein YPP1 at the plasma membrane, which led to the budding of endocytic vesicles via receptor-mediated endocytosis and the subsequent targeting of this form of αSYN to the vacuole for degradation [63]. Furthermore, previous studies by our lab and others have demonstrated that internalized αSYN is secreted from neurons via a process that is modulated by the recycling endosome regulator Rab11a [64,65]. In the case of prion disease, both the normal cellular prion protein (PrPc) and the abnormally folded pathogenic form (PrPsc) are associated with nanovesicles called ‘exosomes’ that are released from non-neuronal and neuronal cells [66,67]. However, the involvement of the exosomal vesicle in αSYN secretion remains unclear. αSYN has been shown to be secreted by the exosome, and exosome-containing conditioned medium can induce neuronal cell death [68,69]. In contrast, we recently demonstrated that extracellular αSYN was mainly detected in the supernatant fraction rather than in the exosome-containing pellets obtained from neuronal culture medium or CSF [64]. Moreover, we found that the perturbation of exosome formation by a DN mutant of vacuolar protein sorting 4 (VPS4) not only interfered with lysosomal targeting of αSYN but also facilitated αSYN secretion [64].

Regardless of the mechanisms involved in αSYN secretion, there is evidence to support the uptake of extracellular αSYN by neighboring cells, which subsequently facilitates aggregate formation. Previous reports have suggested that the internalization of αSYN oligomers may be mediated by the endocytic process; the overexpression of a DN-dynamin effectively reduced the extent of incorporated αSYN aggregates in cultured cell lines [21,39]. Furthermore, the inhibition of endocytosis by monodansylcadaverine and dynasore has also been shown to decrease αSYN uptake both *in vitro* and *in vivo*[22]. Coincubation of αSYN pre-formed fibrils with WGA enhances the αSYN pathology in neuronal cells, indicating adsorptive-mediated endocytosis as the potential mechanism of αSYN internalization [20]. The importance of the endocytic process in the uptake of extracellular αSYN is further supported by our findings showing that genetic as well as pharmacological disruption of the dynamin GTPases through the administration of sertraline, a widely used antidepressant, significantly decreased the internalization and translocation of αSYN. It should also be noted that the concentration of sertraline used in our study (10 μM = 3 μg/ml) is comparable to the concentrations observed to be therapeutically effective within the CSF and brain (2 μg/ml) for its antidepressant effects [70]. In fact, neuropsychiatric manifestations such as depression, apathy, and anxiety are frequently encountered as non-motor symptoms in PD patients [71,72]. SSRIs are currently used as a first-line therapy for PD-associated depression [73]. Thus, the identification of novel therapeutic uses for sertraline not only provides a strategy focused on the prevention of the pathological propagation of αSYN but also has the advantage of utilizing time-tested drugs for the benefit of patients. A recent study has shown that the first SSRI, fluoxetine, ameliorated behavioral and neuropathological deficits in an MSA mouse model [74]. They concluded that the protective effect of fluoxetine might be attributed to the increased level of neurotrophic factors and/or the activation of the ERK signaling pathway; however, the reduction of αSYN-propagation through the inhibition of dynamin may be another underlying mechanism. Indeed, tricyclic antidepressants, which are also known to inhibit dynamin GTPases, have been shown to slow disease progression in PD [41,75]. In the case of MSA, a German group has reported the effectiveness of paroxetine in a small, short-term trial with 19 MSA patients [76]. In this study, motor disabilities and dysarthria were significantly improved compared with the placebo group. It is also interesting to note that paroxetine may prevent the glottis stenosis in MSA patients [77]. Further investigations with larger samples are necessary to assess the long-term safety and effectiveness of SSRI treatment in MSA. We are currently awaiting for the outcome of a double-blind, multicenter trial using fluoxetine for the treatment of MSA (MSA-Fluox) being conducted in France [78].

In summary, we demonstrated that αSYN was taken up by neuronal and oligodendroglial cells, assembled into HMW oligomers, and formed cytoplasmic inclusions. Furthermore, we have provided evidence that αSYN uptake by neuronal and oligodendroglial cells is regulated by dynamin GTPases, which implies a role for the endocytic process in this activity. The importance of the vesicular trafficking machinery in the pathogenesis of PD is also highlighted by recent findings that a mutation in the *VPS35* gene, which encodes a retromer complex involved in the retrograde transport of proteins from the endosome to the trans-Golgi network, can cause late-onset familial PD [79-81]. Furthermore, the prevention of αSYN-mediated pathology by sertraline is a potentially promising method for the treatment of PD and other synucleinopathies. Thus, defining the precise mode of intercellular αSYN transmission will shed light on the pathogenic mechanisms involved in synucleinopathies, and this research may pave the way for the identification of novel targets for therapeutic intervention in other neurodegenerative diseases.

## Methods

### Plasmid construction and preparation

For the bacterial expression of the GST-αSYN fusion protein, human αSYN cDNA was subcloned into the *Sal*I and *Not*I restriction sites of the pGEX6P-1 vector (GE Healthcare Life Sciences, Piscataway, NJ), which encodes an N-terminal GST fusion tag that is cleavable by a human rhinovirus 3C proteinase recognition site (Leu-Glu-Val-Leu-Phe-Gln-Gly-Pro, referred to as the PreScission® site). An N-terminal mCherry-tagged human wt-αSYN cDNA was introduced into the *Kpn*I and *Xho*I sites of the pcDNA3.1+ expression vector (Life Technologies/Invitrogen, Carlsbad, CA). The pEGFP-C1 eukaryotic expression plasmids (Clontech, Mountain View, CA) encoding the EGFP-tagged human wt and DN mutant K44A dynamin 1 were kindly provided by Dr. Hiroshi Miyoshi at the St. Marianna University School of Medicine in Kawasaki, Japan. Plasmid DNA used for transfection was prepared with the Genopure Plasmid Maxi Kit (Roche, Basel, Switzerland). The fidelity and orientation of the expression constructs were confirmed by restriction digestion and nucleotide sequencing analyses.

### Recombinant α-synuclein purification

The GST-αSYN fusion construct (pGEX6P-1/αSYN) was transformed into the BL21(DE3)pLysS *E. coli* strain for protein expression. The transformed bacteria were grown in LB medium containing 100 μg/ml ampicillin and 35 μg/ml chloramphenicol (for pLysS) at 37°C until reaching an *A*_600_ of 0.4. The bacteria were then cultured for an additional 5 hours following induction with 0.5 mM IPTG. The bacteria were harvested by centrifugation, resuspended in ice-cold PBS (pH 7.4), and disrupted by ultrasonication (Smurt NR-50, Microtec, Chiba, Japan). After removal of the cell debris, the supernatant was loaded onto a glutathione-Sepharose 4B column (GE Healthcare) equilibrated with PBS. The GST-αSYN fusion protein was washed with PBS three times and was then eluted with 10 mM glutathione elution buffer. The final eluate that flowed through the column was collected as the control specimen and was further dialyzed against PBS overnight. Next, the GST tag was cleaved overnight at 4°C on a carousel in the presence of the PreScission® protease (2 units for 100 μg fusion protein, GE Healthcare). After cleavage, the sample was re-loaded onto the glutathione-Sepharose 4B column, and the flowthrough containing the tag-free αSYN was collected. The purity and the identity of the recombinant αSYN were verified by CBB staining and western blot analysis. The recombinant βSYN and γSYN, as well as A30P and A53T mutants of αSYN, were purchased from ATGen Co., Ltd. (Gyeonggi-do, Korea).

### Cell culture and plasmid transfection

Dopaminergic neuronal cells (human SH-SY5Y and rat PC12) and human cells of oligodendroglia-lineage (KG1C and MO3.13) were maintained in Dulbecco’s Modified Eagle’s Medium (DMEM; Life Technologies/GIBCO, Carlsbad, CA) containing 4.5 g/l glucose, 2 mM L-glutamine (Life Technologies) and 10% FBS (PAA Laboratories, Pasching, Austria) at a temperature of 37°C under conditions of humidified 5% CO_2_/air. The SH-SY5Y cells were purchased from American Type Culture Collection (ATCC, Vienna, VA). The PC12 cells were kindly given to us by Dr. Katsutoshi Furukawa, Department of Geriatrics and Gerontology, Tohoku University, Sendai, Japan. The KG1C and MO3.13 cells were purchased from RIKEN Cell Bank (Tsukuba, Japan) and Cosmo Bio (Tokyo, Japan), Respectively. Plasmid DNAs (2 μg DNA for 1.5X10^6^ cells) were introduced into the SH-SY5Y cells using the 4D-Nucleofector™ device with the CA-137 program (LONZA AG, Cologne, Germany). The cells were harvested 48 hours post-transfection unless otherwise stated. For the suppression of dynamin GTPases, cells were treated with 5 μM αSYN for 30 min with varying concentration of sertraline as indicated. Likewise, the SH-SY5Y cells were treated with 5 μM αSYN (for 30 min) 48 hours after transfection with the DN-dynamin1 or the siRNA silencing of dynamin 1. Stable transfection of mcherry-αSYN or EGFP in the cultured cells was achieved using the FuGENE HD® Transfection Reagent (Roche) according to the manufacturer’s instructions. For the stable transfection of mcherry-αSYN or EGFP, the transfected cells were maintained under selective pressure with 300 μg/ml G418 sulfate (InvivoGen, San Diego, CA).

### Primary cortical neuron culture

Primary cultures of rat cortical neurons were prepared according to a previous method, with slight modifications [82]. The dissociated cortical neurons were plated at a density of 0.5 × 10^6^ cells on a poly-D-lysine-coated disc (Sumilon Celldesk LF, Sumitomo Bakelite Co., Ltd., Tokyo, Japan) in a 24-well plate and cultured in Neurobasal A (Life Technologies/GIBCO) medium supplemented with 2% B27 (Life Technologies/GIBCO), 25 mM glutamate, 18 mM glucose and 0.5 mM L-glutamine. Half of the culture medium was replaced with fresh medium excluding glutamate every 3 days. Six days after the initiation of the culture, cells were exposed to 5 μM recombinant αSYN for 24 hours and were then subjected to immunocytochemical analysis.

### siRNA knockdown of endogenous dynamin in SH-SY5Y cells

To suppress endogenous dynamin 1 expression in cultured neuronal cells, siRNA specifically targeting human dynamin 1 (sc-43737) was used; this specific siRNA and a scrambled control siRNA (sc-36869) were purchased from Santa Cruz Biotechnology (Santa Cruz, CA). SH-SY5Y cells in log-phase growth were nucleofected with target-specific or control-scrambled siRNAs (300 nM for 2 × 10^6^ cells) using the 4D-Nucleofector device with the CA-137 program and SF solution (LONZA AG). Seventy-two hours after gene silencing, the cells were harvested and subjected to further studies.

### Co-culture experiments

For the mixed culture of αSYN donor and acceptor cells, SH-SY5Y neuronal cells overexpressing mCherry or mCherry-tagged αSYN (2 × 10^5^ donor cells in a 3.5-cm dish) were co-cultured with neuronal PC12 or oligodendroglial MO3.13 cells stably expressing EGFP (2 × 10^5^ acceptor cells in a 3.5-cm dish) in culture medium with or without 10 μM sertraline for 5 days. Incorporated mCherry-αSYN in the acceptor cells was evaluated using a FluoView™ FV300 confocal microscope system equipped with HeNe-Green (543 nm) and Ar (488 nm) laser units (Olympus, Tokyo, Japan). To quantify the mCherry-positive cytoplasmic inclusions in the acceptor cells, the total number of cells containing aggregates was counted per 250–300 cells from each of five randomly selected fields. From this, the percentages of cells with red-fluorescent inclusions were calculated. Data pooled from four independent experiments were statistically analyzed with the Student's *t*-test. The data were presented as the mean ± standard errors. For the double labeling experiments, the images were collected using a single excitation for each wavelength individually and were then merged using Fluoview image analyzing software (version 4.1, Olympus).

### Cell fractionation and western immunoblot analysis

Mechanically harvested cells were washed twice with ice-cold PBS. Next, the hydrophobic membrane fraction and hydrophilic fraction were prepared using the Mem-PER® Eukaryotic Membrane Protein Extraction Reagent kit (Thermo Scientific, Waltham, MA) according to the manufacturer’s instructions. In some experiments, the cells were pretreated with bafilomycin A1 (0–5 nM for 24 hours) or sertraline (0–10 μM for 30 min) with or without exposure to αSYN. Successful separation of the hydrophilic fraction from the membrane fraction was verified by immunoblot analyses using Abs against the cytosolic Hsp90 and the plasma membrane localized protein Na^+^/K^+^ ATPase α, respectively. In some experiments, cells were directly solubilized in radio-immunoprecipitation assay (RIPA) buffer (1% NP-40, 0.5% deoxycholate, 0.1% SDS, 1 mM EDTA, 10 mM sodium pyrophosphate, 50 mM sodium fluoride, 1 mM sodium orthovanadate, 150 mM sodium chloride, 50 mM Tris–HCl (pH 8.0) plus 1x Cømplete® protease inhibitor cocktail; Roche). Samples containing 50 μg total protein were electrophoresed on SDS-polyacrylamide gels (12.5%) and transferred onto polyvinylidene difluoride (PVDF) membranes (Immobilon-P; Merck Millipore, Billerica, MA). Native-PAGE was performed on 10-20% polyacrylamide gradient gels, according to the standard protocol. In some experiments, the separated proteins were visualized by staining with CBB R-250 (MP Biomedicals, Solon, OH). After blocking with 5% (w/v) nonfat dry milk (Wako Pure Chemical Industries, Ltd., Osaka, Japan) in Tris-buffered saline with 0.1% Tween 20 (TBST), the membranes were incubated with the following Abs: anti-synuclein-1 (63320, 1:1000; BD Bioscience, San Jose, CA), anti-αSYN (#2628, 1:1000; CST, Danvers, MA), anti-GST (#2625, 1:1000: CST), anti-LC3 (PM036, 1:1000; MBL, Nagoya, Japan), anti-caspase 3 (H-277, 1:2000; Santa Cruz), anti-cleaved caspase 3 (Asp 175, 1:1000; CST), anti-dynamin 1 (3G4B6, 1:1000; CST), anti-dynamin 2 (610263, 1:4000; BD Transduction Lab, Franklin Lakes, NJ), anti-dopa decarboxylase (DDC) (AB1569, 1:1000; Millipore), anti-TPPP/p25α (EPR3315, 1:1000; Epitomics, Burlingame, CA), anti-GFP (M048-3, 1:2000; MBL) anti-Na^+^/K^+^ ATPase α (D154-3, 1:20000; MBL), anti-Hsp90 (AC88, 1:2000; Stressgen, Farmingdale, NY), anti-α-tubulin (clone DM1, 1:1000; Sigma) and anti-mCherry (5993, 1:1000; BioVision, Mountain View, CA). The primary antibody labeling was followed by the addition of HRP-conjugated secondary Abs (1:10000; Jackson ImmunoResearch, West Grove, PA). The bands were visualized with the Luminata™ Forte Western HRP substrate (Millipore), and the images were captured using the LAS-3000 mini image analyzer (Fujifilm, Tokyo, Japan). Immunoblotting was performed at least three times. In some experiments, the blots were scanned and densitometric analyses were performed using Image J ( http://www.rsb.info.nih.gov/ij/) [83]. The intensity unit of the αSYN monomer was divided by that of Hsp90. Data are expressed as the mean ± standard errors.

### Immunofluorescence confocal microscopy

Cells were fixed in 4% (w/v) paraformaldehyde in PBS for 30 min and then permeabilized with 0.5% Triton X-100 in PBS for 5 min. After blocking with 3% normal goat serum (Wako Pure Chemical Industries) in PBS for 30 min, the cells were incubated with the following primary antibodies: anti-synuclein-1 (1:1000; BD Bioscience), anti-αSYN (#2628, 1:2000; CST), anti-Serine 129 phospho-αSYN (EP1526Y, 1:1000; Epitomics), anti-γ-tubulin (GTU-88, 1:4000; Sigma, St. Louis, MO), anti-peripherin (AB1530, 1:1000; Millipore-Chemicon), anti-vimentin (V9, 1:500; Sigma), anti-ubiquitin (P4D1, 1:1000; Santa Cruz), anti-MAP2 (#4542, 1:1000; CST), anti-TPPP/p25 (EPR-3315, 1:1000; Epitomics), anti-Rab5A Ab (S-19, 1:1000; Santa Cruz) and anti-Lamp-1 (H4A3, 1:1000; DSHB, University of Iowa, USA). For thioflavin S staining, the coverslips were immersed in 0.03% (w/v) thioflavin S (Sigma) solution for 5 min and were then extensively washed with 70% ethanol. Positive immunostaining was detected after a 1-hour incubation with Alexa 488- and Alexa 568-conjugated secondary Abs (1:4000; Life Technologies/Molecular Probes, Carlsbad, CA). Nuclei were counterstained with TO-PRO3 iodide (1:1000; Molecular Probes) and were pseudocolored in blue. The fluorescent images were analyzed with the FluoView FV300 confocal laser scanning microscope system (Olympus). The crossed lines indicate the positions of the xz- and yz-planes. To quantify the αSYN-immunopositive inclusions, the total number of cells with any aggregates was counted across 250–300 cells in five randomly chosen fields. From this, the percentages of cells with inclusions were calculated. Pooled data from four independent experiments were statistically analyzed using the Student's *t*-test. The data are expressed as the mean ± standard errors.

## Abbreviations

αSYN: α-synuclein; βSYN: β-synuclein; γSYN: γ-synuclein; LB: Lewy body; PD: Parkinson’s disease; GCI: Glial cytoplasmic inclusion; MSA: Multiple system atrophy; SDS-PAGE: Sodium dodecyl sulfate-polyacrylamide gel electrophoresis; IPTG: Isopropyl β-D-1-thiogalactopyranoside; E. *coli*: *Escherichia coli*; CBB: Coomasie brilliant blue; HMW: High molecular weight; ER: Endoplasmic reticulum; TPPP/p25α: Tubulin polymerization promoting protein/p25α; WGA: Wheat germ agglutinin; PrP: Prion protein; VPS4: Vacuolar protein sorting 4; SSRI: Selective serotonin-reuptake inhibitor; EGFP: Enhanced Green Fluorescence Protein; wt: wild-type; DN: Dominant-negative; DMEM: Dulbecco’s Modified Eagle’s Medium; siRNA: Small interference RNA; RIPA: Radio-immunoprecipitation assay; PVDF: Polyvinylidene difluoride; TBST: Tris-buffered saline with 0.1% Tween 20; Ab: Antibody; DDC: Dopa decarboxylase.

## Competing interests

The authors declare that they have no competing interests.

## Authors’ contributions

MK, TH, TB, and EM performed the experiments. NS, AK, and AT analyzed the data. FCF, TS, MA, and YI contributed reagents/materials. MK and TH designed the study and wrote the paper. AT is the principal investigator. All authors read and approved the final manuscript.

## Supplementary Material

Additional file 1 **Figure S1.** Analyses of internalized α-synuclein monomer in exposed cells. A, The densitometric analysis of monomeric αSYN in hydrophilic fraction prepared from αSYN-exposed SH-SY5Y (a and c) and KG1C cells (b). The intensity units of the αSYN monomer were normalized by dividing them by that of Hsp90. Data are expressed as the mean ± standard errors. B, The GST-tagged αSYN (5 μM) in the culture medium was time-dependently detected and shown to form HMW GST-immunopositive oligomers and the HMW smear mainly in the hydrophilic fraction of the SH-SY5Y cells, demonstrating that the extracellular αSYN was internalized and oligomerized in the exposed cells. All immunoblottings were performed four times. Click here for file

Additional file 2 **Figure S2.** The difference in α-synuclein internalization behavior among mammalian synuclein-family proteins. To evaluate the difference in the internalization behavior among the synuclein-family proteins, SH-SY5Y cells exposed to 5 μM α-, β- and γ-SYN, were fractionated and subjected to immunoblot analyses (right panel). Note that αSYN was exclusively internalized into the SH-SY5Y cells, whereas β- and γ-SYN were not. Furthermore, the A30P and A53T mutations in αSYN strongly augmented the formation of the SDS-stable oligomers in the hydrophilic fraction when compared to those observed in the wt-αSYN-exposed cells. The specificity and sensitivity of each synuclein Ab were verified by immunoblotting using the lysates of HEK293T cells overexpressing Myc-tagged α-, β- and γ-SYN, respectively (left panel). Representative immunoblots from three independent experiments are shown. Click here for file
