# Supplementary material for: Suppression of dynamin GTPase decreases α-synuclein uptake by neuronal and oligodendroglial cells: a potent therapeutic target for synucleinopathy
Source: Mol Neurodegener. 2012 Aug 14;7:38. doi: 10.1186/1750-1326-7-38 (PMC3479026; doi:10.1186/1750-1326-7-38)
Supplement: Additional file 1 — Figure S1. Analyses of internalized α-synuclein monomer in exposed cells. A, The densitometric analysis of monomeric αSYN in hydrophilic fraction prepared from αSYN-exposed SH-SY5Y (a and c) and KG1C cells (b). The intensity units of the αSYN monomer were normalized by dividing them by that of Hsp90. Data are expressed as the mean ± standard errors. B, The GST-tagged αSYN (5 μM) in the culture medium was time-dependently detected and shown to form HMW GST-immunopositive oligomers and the HMW smear mainly in the hydrophilic fraction of the SH-SY5Y cells, demonstrating that the extracellular αSYN was internalized and oligomerized in the exposed cells. All immunoblottings were performed four times. [file 1750-1326-7-38-S1.ppt]

## Slide 1
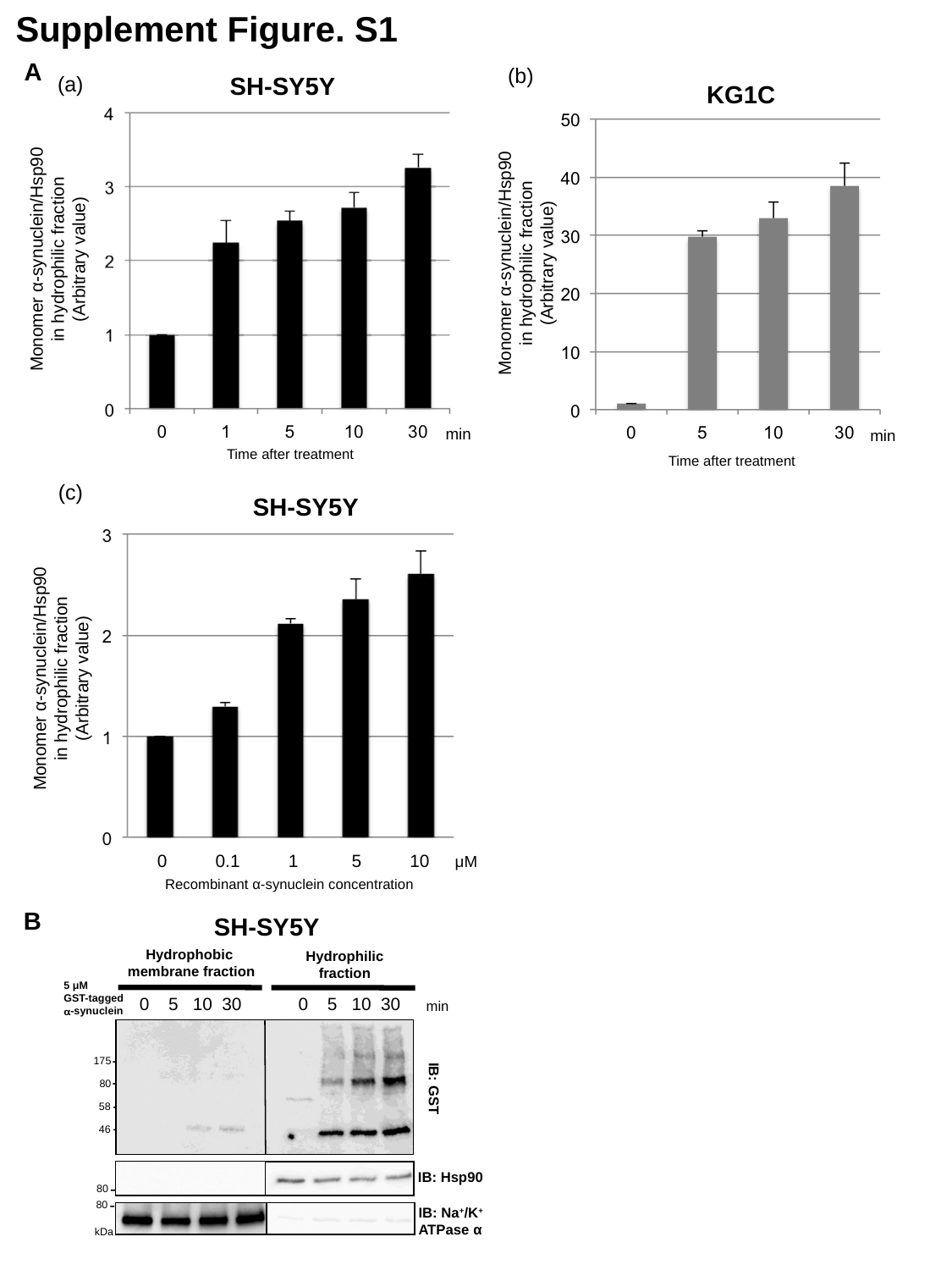

Supplement Figure. S1
A
(b)
(a)
SH-SY5Y
KG1C
Monomer α-synuclein/Hsp90 in hydrophilic fraction
(Arbitrary value)
Monomer α-synuclein/Hsp90 in hydrophilic fraction
(Arbitrary value)
min
min
Time after treatment
Time after treatment
(c)
SH-SY5Y
Monomer α-synuclein/Hsp90 in hydrophilic fraction
(Arbitrary value)
0 0.1 1 5 10
μM
Recombinant α-synuclein concentration
B
SH-SY5Y
Hydrophobic
membrane fraction
Hydrophilic fraction
5 μM
GST-tagged
α-synuclein
0 5 10 30
0 5 10 30
min
175
80
IB: GST
58
46
IB: Hsp90
80
80
IB: Na+/K+
ATPase α
kDa
