# Supplementary material for: Suppression of dynamin GTPase decreases α-synuclein uptake by neuronal and oligodendroglial cells: a potent therapeutic target for synucleinopathy
Source: Mol Neurodegener. 2012 Aug 14;7:38. doi: 10.1186/1750-1326-7-38 (PMC3479026; doi:10.1186/1750-1326-7-38)
Supplement: Additional file 2 — Figure S2. The difference in α-synuclein internalization behavior among mammalian synuclein-family proteins. To evaluate the difference in the internalization behavior among the synuclein-family proteins, SH-SY5Y cells exposed to 5 μM α-, β- and γ-SYN, were fractionated and subjected to immunoblot analyses (right panel). Note that αSYN was exclusively internalized into the SH-SY5Y cells, whereas β- and γ-SYN were not. Furthermore, the A30P and A53T mutations in αSYN strongly augmented the formation of the SDS-stable oligomers in the hydrophilic fraction when compared to those observed in the wt-αSYN-exposed cells. The specificity and sensitivity of each synuclein Ab were verified by immunoblotting using the lysates of HEK293T cells overexpressing Myc-tagged α-, β- and γ-SYN, respectively (left panel). Representative immunoblots from three independent experiments are shown. [file 1750-1326-7-38-S2.ppt]

## Slide 1
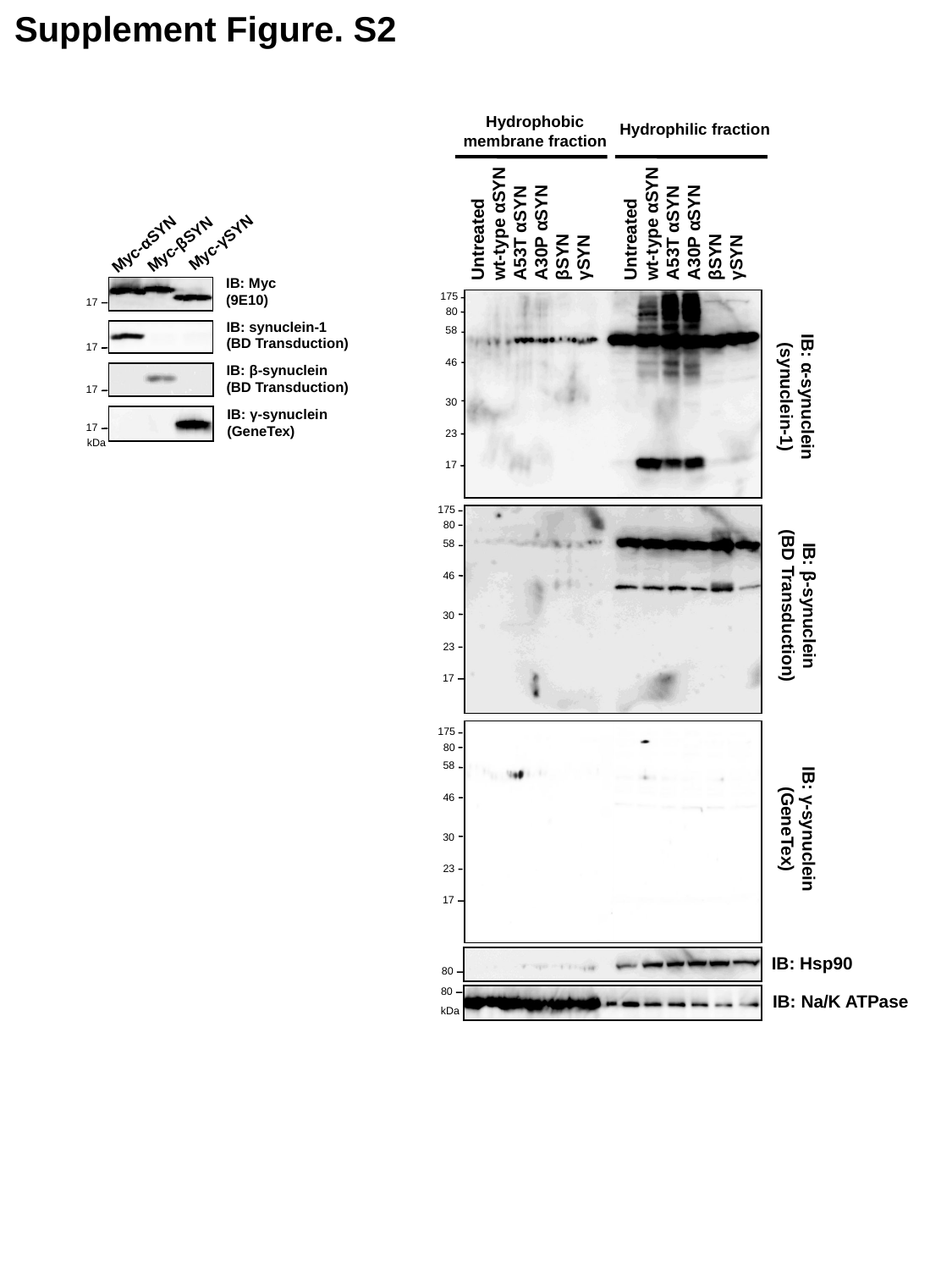

Supplement Figure. S2
Hydrophobic membrane fraction
Hydrophilic fraction
Untreated
wt-type αSYN
A53T αSYN
A30P αSYN
βSYN
γSYN
Untreated
wt-type αSYN
A53T αSYN
A30P αSYN
βSYN
γSYN
Myc-γSYN
Myc-βSYN
Myc-αSYN
IB: Myc
(9E10)
175
17
80
IB: synuclein-1
(BD Transduction)
58
17
46
IB: β-synuclein
(BD Transduction)
IB: α-synuclein
(synuclein-1)
17
30
IB: γ-synuclein
(GeneTex)
17
23
kDa
17
175
80
58
46
IB: β-synuclein
(BD Transduction)
30
23
17
175
80
58
46
IB: γ-synuclein
(GeneTex)
30
23
17
IB: Hsp90
80
80
IB: Na/K ATPase
kDa
